# The vectorial transport of salts and water is crucial for respiratory epithelial cell lines

**DOI:** 10.1186/s12931-015-0235-1

**Published:** 2015-06-12

**Authors:** Khaled Khoufache

**Affiliations:** Research Center, Saint-François d’Assise Hospital, Centre Hospitalier Universitaire de Québec (CHUQ), Québec City, QC Canada

**Keywords:** Airway epithelial model, Mucociliary differentiation, Polarization, Trans epithelial resistance, Trans epithelial ionic flux

## Abstract

Primary culture of respiratory epithelial cells is useful to study the pathophysiology of respiratory diseases. However, such primary culture has been very limited because of its high dependence on the availability of biopsies and the long time required to reach confluence. Therefore, cell lines are an alternative to primary cultures because they reach confluence faster and some can maintain their differentiation abilities. However, unlike primary cultures and native tissues just some cell lines are able to polarize, with normal channel functionality and transepithelial ionic flux.

## Dear Editor

I read with great enthusiasm the recent article written by Matthew Walters and colleagues in BMC Respiratory Research [1]. The authors reported the establishment of a basal cell line, BCi NS1.1, from respiratory epithelial basal cells of healthy non-smokers. The original cells were immortalized using a recombinant virus carrying the human telomerase gene. The established cell line maintained the properties of the original cells 30 even after 40 passages. Moreover, a mucociliary phenotype (positivity for MUC5AC, MUC5B, TFF3, CC10, DNAI1, and FoxJ1) was obtained by culture at the liquid air-interface. In addition, confluence of the cultures was confirmed by trans epithelial resistance (Rte) (57 ± 4 Ω × cm^2^ at day 10, 165 ± 67 Ω × cm^2^ at day 14, 1312 ± 281 Ω × cm^2^ at day 28, and 1563 ± 86 Ω × cm^2^ at day 40). Therefore, the authors concluded that the BCi NS1.1 cell line accurately mimics the airway epithelium, and can be considered as a model to study the interactions with environmental stimuli, cytokines, cigarette smoke or as a target for assessing of pharmacologic agents. However, studies of such interactions with the environment or drugs require not only a 100 % confluent culture but also polarization. The authors examined the Rte but this parameter is not sufficient to make conclusions about the polarization status of the cell line. Additionally, the Rte was 57 ± 4 Ω × cm^2^ (day 10) and 165 ± 67 Ω × cm^2^ (day 14) [2–5]. Moreover, this cell line requires more than 20 days to obtain Rte, which is comparable to the native epithelium [5]. Furthermore, confluence and the differentiation capacity of the monolayer do not necessarily indicate functionality, because a biological process called trans epithelial vectorial transport of salts and water must be present. In fact, normal flux transport indicates normal expression and functions of membrane-bound ion channels such as NaCl-, K+, and Na + and the cystic fibrosis transmembrane conductance regulator (CFTR) [4, 6–9]. Each of these channels is crucial because dysfunction of one can cause severe pathologies [10]. A mutation in CFTR causes cystic fibrosis, suggesting the importance of this channel protein [11]. However, it has been documented that CFTR is not expressed in human airway basal cells [12]. Moreover, a previous study has shown that primary cystic fibrosis cell culture and some cystic fibrosis cell lines, secretes Cl in response to agonists [13]. That why it is important to analyze the transepithelial ionic flux. For this purpose, two parameters can be measured: (i) the potential difference (mV) and (ii) the short circuit current (Isc) (μA/cm^2^). Similar to native epithelium, passage 2 and 3 tracheobronchial epithelial cell cultures show Rte (500–800 Ω × cm^2^), but the equivalent Isc is much lower than that in native tissue (3 μA/cm2) [5, 14]. Furthermore, the Rte and Isc decrease to 100 Ω × cm^2^ and 2 μA/cm, respectively, at passage 4. In another study, human airway epithelial cells were successfully passaged up to six times with an Rte of 1000–4000 Ω × cm^2^ and Isc of 5 μA/cm [15]. Moreover, the results indicated few ciliated cells (only 10 %), whereas ciliated cells comprise 60–90 % of the surface of normal respiratory epithelium. In addition to maintaining barrier integrity, ciliated cells are important for absorption and secretion of electrolytes, which generate potential difference [16, 17]. Therefore, I believe that differentiation and cell polarization are inseparable parameters. Consequently, checking the new cell line for its vectorial transport of salts and water would be a useful addition.

## Abbreviations

BCi NS1.1: Clone basal cell immortalized-nonsmoker 1
KRT5: Keratin 5
TP63: Tumor protein p63
TFF3: Trefoil factor 3
MUC5AC: Mucin 5, subtypes A and C
MUC5B: Mucin 5, subtype B
CC10: Clara cell protein 10
DNAI1: Dynein, axonemal, intermediate chain 1
FoxJ1: Forkhead box J1
